# Overcoming anaphylactic severe cow’s milk allergy with slow and low-dose oral immunotherapy: A 10-year path to tolerance

**DOI:** 10.5415/apallergy.0000000000000248

**Published:** 2025-12-02

**Authors:** Shungo Yamamoto, Kiwako Yamamoto-Hanada, Tatsuki Fukuie, Yukihiro Ohya

**Affiliations:** 1 Allergy Center, National Center for Child Health and Development, Tokyo, Japan; 2 Department of Occupational and Environmental Health, Graduate School of Medical Sciences, Nagoya City University, Aichi, Japan; 3 Division of General Allergy, Bantane Hospital, Fujita Health University, Aichi, Japan

**Keywords:** Anaphylaxis, cow’s milk allergy, oral immunotherapy

## Abstract

Cow’s milk allergy (CMA) is a common pediatric food allergy that can cause significant nutritional and quality-of-life challenges. Severe cases, characterized by high milk-specific IgE levels and a history of anaphylaxis, rarely develop natural tolerance. Oral immunotherapy (OIT) is generally avoided in such high-risk patients due to the risk of serious allergic reactions, leaving strict avoidance as the standard treatment. However, prolonged avoidance may delay tolerance acquisition and increase psychosocial burden.

We report a female patient with severe CMA and multiple food allergies who underwent a carefully tailored, ultra-low-dose OIT over 10 years. At treatment initiation, her milk-specific IgE was >100 kUA/L, and an oral challenge with 1.1 mL of milk induced anaphylaxis. The OIT protocol started with a dose well below her reaction threshold and increased gradually, resulting in no serious adverse events. Over time, she achieved tolerance to 200 mL of pure milk and resumed unrestricted consumption of milk and other previously avoided foods such as egg and wheat.

This is the first report of successful long-term ultra-low-dose OIT in a highly sensitized child with severe CMA, showing that slow, cautious escalation can safely induce unrestricted intake even in patients previously deemed unsuitable for OIT.

Given the limitations of biologics and emergency care availability worldwide, this low-risk, home-based protocol using locally available foods offers a feasible and affordable approach for managing severe food allergies globally. Such individualized, sustained OIT may improve long-term outcomes and quality of life for children with severe food allergies.

## 1. Introduction

Cow’s milk allergy (CMA) is a common pediatric food allergy [1] that can significantly impair nutrition and quality of life. While many children outgrow CMA, those with severe IgE-mediated reactions—marked by high milk-specific IgE levels and a history of anaphylaxis—are far less likely to achieve natural tolerance. In such high-risk cases, oral immunotherapy (OIT) is generally not recommended due to the substantial risk of inducing serious allergic reactions, including anaphylaxis. Consequently, complete avoidance remains the standard of care. However, prolonged avoidance may delay tolerance development and lead to psychosocial burden.

Here, we report an instructive and successful case of a highly sensitized child with severe CMA who no longer requires dietary avoidance of milk through a carefully tailored, ultra-low-dose OIT protocol [2, 3] below the threshold (avoiding up-dosing above the reaction threshold) conducted over 10 years. In addition to cow’s milk, the patient had multiple sensitizations and food allergies, including egg, wheat, and peanuts—yet was ultimately able to reintroduce all previously avoided foods. She transitioned into adolescence with no dietary restrictions, representing a notable outcome for a child initially deemed ineligible for OIT. This case underscores the potential for individualized, long-term desensitization strategies to achieve avoidance-free eating, even in children with multiple, severe food allergies.

## 2. Case report

A female patient was diagnosed with CMA at 7 months after experiencing immediate-type reactions in infancy. At age 4, she was referred to our center with ongoing elimination of milk, egg, and wheat. She had multiple food sensitizations, food allergies, and comorbid asthma, atopic dermatitis, allergic rhinitis, and conjunctivitis (Table 1). Atopic dermatitis was managed with proactive therapy by topical corticosteroids, asthma with a leukotriene receptor antagonist and inhaled corticosteroids (no exacerbations after age 7), and allergic rhinitis and conjunctivitis as needed with antihistamines. Sublingual immunotherapy for dust mite and Japanese cedar pollen was considered as a treatment option. The patient did not wish to undergo sublingual immunotherapy because the symptoms were well controlled with symptomatic treatment and environmental adjustments. At age 7, total IgE was 4,546 IU/mL, and milk-specific IgE was >100 kUA/L, and 1.1 mL oral milk challenge induced anaphylaxis.

**Table 1.** Characteristics of initial blood tests

Total IgE	17800	IU/mL
Specific IgE (Immunocap)		
Cow’s milk	90.6	UA/mL
Casein	>100	UA/mL
Egg white	50.8	UA/mL
Ovomucoid	56.7	UA/mL
Wheat	64.6	UA/mL
Gluten	82.9	UA/mL
Peanut	20	UA/mL
Sesame	23.7	UA/mL
Buckwheat	21.8	UA/mL
Shrimp	62.2	UA/mL
Crab	68.7	UA/mL
Mackerel	3.35	UA/mL
Kiwi	4.61	UA/mL
Dermatophagoides pteronyssinus	> 100	UA/mL
Staphylococcus aureus B	15.4	UA/mL

**Figure 1.**
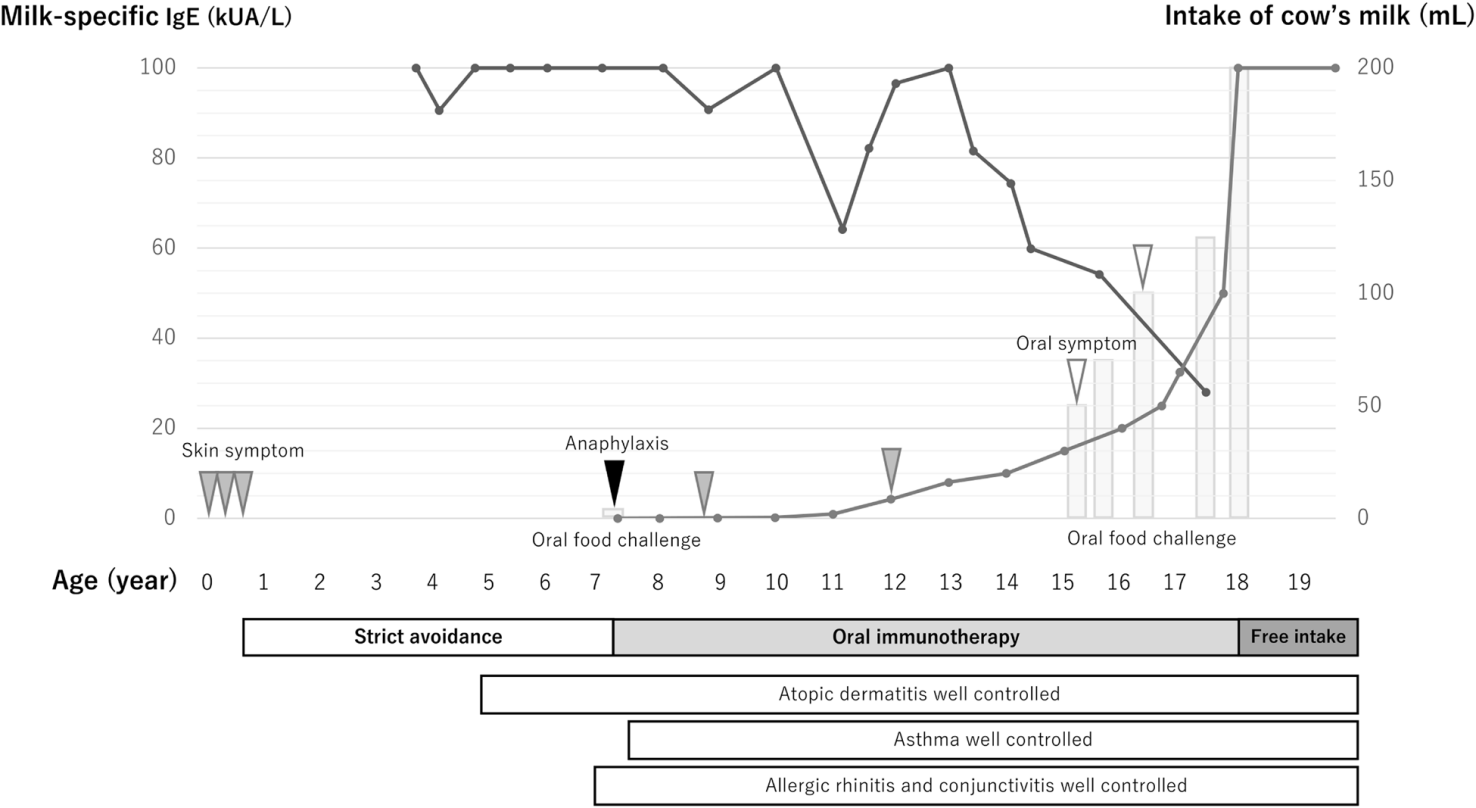
Clinical course. This figure shows longitudinal changes in milk-specific IgE levels and cow’s milk intake in a patient with persistent cow’s milk allergy. Milk-specific IgE levels exceeding 100 kUA/L are displayed as 100. Periods of free milk consumption are represented as an intake of 200 mL. Gray bars represent the amount of cow’s milk ingested during oral food challenges. Black, gray, and white arrowheads indicate episodes of anaphylaxis, skin symptoms, and oral symptoms, respectively.

OIT was initiated with 0.01 mL of diluted milk (1 mL of 1:100 dilution), below her reaction threshold, and administered at least 3 times per week at home. Dose escalation occurred at intervals of several months based on consistent intake without reactions. By year 1, she tolerated 0.03 mL. Facial flushing occurred at 0.22 mL but did not recur. Up-dosing proceeded cautiously. By year 3, she tolerated 0.35 mL; by year 4, 1 mL; by year 5, 9 mL with only transient pruritus; by year 6, 16 mL. She began ingesting processed milk-containing foods within tolerated levels, improving her dietary variety and adherence. Around this time, milk-specific IgE levels began to decline. Between years 8 and 10, she completed staged challenges (50, 70, 100, 125, and 200 mL), experiencing only mild oral symptoms at intermediate doses. At year 10, she passed the 200 mL challenge without symptoms and began freely consuming milk and dairy products at home (Fig. 1).

In addition to milk, she successfully reintroduced previously avoided or sensitized foods—such as egg and wheat—without restrictions (Table 2).

**Table 2.** Food intake history

	Progress up to age 17	At age 19 years
Egg white	At 6 months of age, the patient consumed bread porridge once and then discontinued it. At 5 years old, she experienced anaphylaxis with cutaneous and respiratory symptoms at an OFC dose of 3.4 g egg white. Egg white-specific IgE was 55.0 UA/mL, and ovomucoid-specific IgE was 84.8 UA/mL. At 6 years old, OIT was started at 1 g of egg white. Egg white-specific IgE was 59.3 UA/mL, and ovomucoid-specific IgE was over 100 UA/mL, but both gradually decreased thereafter. At 8 years old, she passed the OFC at 35 g egg white. Egg white-specific IgE was 13.4 UA/mL, and ovomucoid-specific IgE was 25.9 UA/mL. Afterward, she consumed bread at home prepared with various heating methods.	Cooked egg-free
Wheat	At 6 months of age, only ate bread porridge once, then stopped, and wheat was avoided due to positive sensitization. At 5 years of age, she passed OFC at 3.5 g udon. Then, she increased her intake at home. Wheat-specific IgE was 59.5 UA/mL. At 7 years of age, she could eat wheat freely, and wheat-specific IgE was 30.5 UA/mL.	Free
Peanut	Peanut was avoided due to positive sensitization. At 6 years old, she passed OFC 7.4 g peanuts, and peanut-specific IgE was 11.9 UA/mL. At 9 years old, she passed OFC at 8.4 g peanuts, and peanut-specific IgE was 8.51 UA/mL.	Free
Almond	Almond was avoided due to positive sensitization. At 10 years old, she passed OFC at 9.6 g almonds, and almond-specific IgE was 2.52 UA/mL.	Free
Walnut	Walnut was avoided due to positive sensitization. At 10 years old, she passed OFC at 8.0g walnuts, and walnut-specific IgE was 3.53 UA/mL.	Free
Buckwheat	Buckwheat was avoided due to positive sensitization. She started with a small amount at home and gradually increased.	Free
Kiwi	Kiwi was avoided due to positive sensitization. She started with a small amount at home and gradually increased.	Free

“Free” indicates that the food can be consumed without restriction and without allergic symptoms.

OFC, oral food challenge; OIT, oral immunotherapy.

## 3. Discussion

This is the first known report of a child with severe CMA—defined by milk-specific IgE >100 kUA/L and anaphylaxis to 1.1 mL—achieving unrestricted intake through long-term, slow, ultra-low-dose OIT. Past studies indicated that children with high IgE levels and a history of anaphylaxis are at risk for persistent CMA [4]. Traditional OIT protocols often involve higher starting doses and rapid escalation beyond the reaction threshold, resulting in increased adverse events with high anaphylaxis risk and high dropout rates [5, 6].

In contrast, our protocol prioritized safety by initiating doses well below the reaction threshold and escalating gradually over years [2, 3]. The initial dose is set below one-tenth of the eliciting dose, with the maintenance dose remaining under the reaction threshold. After several months of stable intake, we do an oral food challenge to assess if the tolerated dose is increased, followed by gradual home up-dosing below the confirmed tolerance level (non-reaction dose). Home intake is recommended at least 3 times per week. Patients are instructed on emergency management, including epinephrine autoinjector use and prompt medical evaluation. Comorbid allergic diseases are managed comprehensively as part of a holistic approach. At our hospital, all patients, including severe cases, are eligible for OIT. In this case, treatment began at one-hundredth of the eliciting dose, with maintenance gradually increased while confirming the absence of symptoms. No adrenaline use or serious reactions occurred. OIT prognosis is influenced by age, IgE level, and eczema [2]. This case had high milk-specific IgE, anaphylaxis history, and complete avoidance—factors associated with no natural tolerance in school-age children [7]. These risk factors suggest minimal likelihood of spontaneous tolerance acquisition in this patient. The prolonged treatment duration likely reflects the initial disease severity requiring gradual desensitization. While long-term treatment complicates distinguishing OIT-induced from natural tolerance, free intake achievement only after systematic dose escalation strongly supports the role of OIT. In addition, even mild oral allergy symptoms induced during the challenge test were accompanied by a clear increase in urinary prostaglandin D₂ metabolite (PGDM) levels [8]. PGDM is a marker that increases during immediate-type food allergic reactions [9, 10], indicating that an allergic response was indeed occurring in the body. We believe that successful OIT requires conducting treatment without inducing any allergic symptoms, including oral allergy syndrome (OAS). Therefore, we sometimes refrain from strongly recommending sublingual immunotherapy for inhalant allergens that inevitably cause OAS, considering the possibility that it may adversely affect OIT treatment.

Food allergy affects children worldwide—not only in developed countries but also in low- and middle-income nations. In such settings, the cost of treatment and access to specialized medical care may pose significant barriers. Therefore, effective and sustainable treatment strategies must be affordable, low-cost, locally accessible, and feasible for home use. Although the use of biologics such as omalizumab has shown promise [11], their high cost limits applicability in resource-limited settings. Furthermore, many countries still lack access to adrenaline autoinjectors and emergency care, rendering high-risk OIT protocols unfeasible. A protocol that avoids anaphylaxis, does not require adrenaline, can be implemented safely at home, and uses locally available foods is urgently needed. The gradual low-dose OIT protocol used in this case may serve as a viable model for such a global application.

A limitation is that sustained unresponsiveness (SU) was not evaluated. Guidelines define SU as tolerance after a period of avoidance, but definitions vary regarding duration (2–8 weeks, occasionally 6 months) and dose. Short-term SU does not ensure long-term unrestricted intake. Moreover, studies such as Peanut Oral Immunotherapy: Safety, Efficacy, Discovery (POISED) demonstrate that stopping OIT can increase the risk of re-sensitization or relapse [12]. Thus, interrupting allergen ingestion solely to assess SU may expose patients to harm. From the patient’s perspective, the meaningful patient-oriented outcome is unrestricted daily consumption, not transient SU. We therefore consider unrestricted intake to be the most relevant treatment goal.

## 4. Conclusion

Families of children with severe food allergies seek treatment options that aim for long-term resolution, not just indefinite avoidance. This case highlights the potential for even highly sensitized children to achieve unrestricted intake through carefully tailored, low-risk interventions. Notably, children with more severe phenotypes are less likely to outgrow their allergies through natural resolution alone. Therefore, proactive strategies that promote immune tolerance—such as slow, sustained OIT—may be essential to alter the natural course of the disease and improve long-term outcomes.

## Acknowledgments

Thank you to all the staff at our institute who cared for this patient.

## Conflicts of interest

The authors have no financial conflicts of interest.

## Author contributions

Shungo Yamamoto and Kiwako Yamamoto-Hanada contributed to the study design, prepared study results, and led the drafting of the manuscript. All co-authors contributed to the interpretation of the findings. All co-authors contributed to revising the manuscript and approved the final version.
